# Influence of Atopic Dermatitis on the Dermoscopic Phenotype of Positive Patch Test Reactions: A Prospective Comparative Study

**DOI:** 10.3390/life16040663

**Published:** 2026-04-14

**Authors:** Anna Zaryczańska, Małgorzata Sokołowska-Wojdyło, Roman J. Nowicki, Magdalena Trzeciak

**Affiliations:** 1 Department of Dermatology, Venereology and Allergology, Medical University of Gdańsk, 80-214 Gdańsk, Poland; 2 Department of Dermatology, Venereology and Allergology, University Clinical Centre, 80-214 Gdańsk, Poland

**Keywords:** atopic dermatitis, patch test reactions, dermoscopy, allergic contact dermatitis, contact allergy, skin barrier dysfunction, inflammatory dermatoses, non-invasive diagnostics

## Abstract

Atopic dermatitis (AD) may alter the morphology of clinically positive allergic patch test reactions due to epidermal barrier dysfunction and a chronic inflammatory background, thereby complicating routine clinical interpretation. This prospective observational study aimed to evaluate the dermoscopic features of clinically positive allergic patch test reactions and compare their phenotypic expression in patients with and without concomitant AD. Consecutive patients undergoing routine patch testing were screened, and clinically positive reactions (1+, 2+, or 3+ according to ICDRG criteria) were subjected to dermoscopic assessment at scheduled readings. A total of 160 participants were included. Patch test reactions in patients with AD exhibited a consistent dermoscopic pattern, characterized by an increased frequency of perifollicular erythema, yellowish areas, and pigment residuals, whereas homogeneous erythema demonstrated limited discriminatory value in both pediatric and adult populations. Yellowish areas were additionally associated with greater AD severity. These findings suggest that AD may modify the phenotypic expression of clinically positive allergic patch test reactions, particularly when evaluated using dermoscopy. This non-invasive diagnostic tool may provide adjunctive morphological support for patch test interpretation, especially in equivocal cases.

## 1. Introduction

Allergic contact dermatitis (ACD) is a common inflammatory dermatosis caused by cutaneous exposure to low-molecular-weight chemicals (haptens) that trigger a T-cell-mediated delayed-type hypersensitivity response. Patch testing remains the reference standard for the diagnosis of ACD and is routinely used to identify clinically relevant contact sensitization [1,2]. Despite its pivotal role in dermatological practice, patch test interpretation still depends largely on visual inspection and semi-quantitative grading. This approach is inherently subjective and particularly vulnerable to inter-observer variability in weak or doubtful reactions, in which subtle epidermal and vascular changes may escape reliable clinical recognition [3]. Improving the assessment of such borderline responses is therefore of direct clinical relevance, as diagnostic uncertainty can influence conclusions regarding allergen relevance, patient counseling, avoidance recommendations, and long-term disease management.

Atopic dermatitis (AD) represents a particularly challenging setting for patch test interpretation. The disease is characterized by epidermal barrier dysfunction and immune dysregulation, with type 2-predominant inflammation that alters keratinocyte differentiation, promotes spongiosis, and modifies local cutaneous reactivity [4,5]. Structural barrier abnormalities, including filaggrin deficiency and disturbances in epidermal lipid composition, facilitate the penetration of environmental triggers such as haptens and irritants and contribute to the persistence of a chronically activated inflammatory milieu [6,7]. In routine clinical practice, baseline erythema, lichenification, excoriations, and pigmentary alterations commonly observed in AD may overlap with or obscure patch test reactions. Consequently, patch test morphology in AD may reflect both allergen-specific delayed hypersensitivity and background atopic inflammation, increasing the risk of overinterpretation as well as underrecognition [8,9,10].

Non-invasive imaging techniques may help address these diagnostic limitations by providing additional micro-morphological information beyond naked-eye examination. Dermoscopy and high-resolution videodermoscopy allow rapid in vivo assessment of epidermal and superficial dermal structures, including vascular architecture, scaling, follicular involvement, spongiosis-related features, and pigment distribution [11,12]. In the context of patch testing, dermoscopy has been proposed as a useful adjunctive tool for the evaluation of equivocal reactions and may assist in distinguishing allergic from irritant response patterns [10,11]. However, the current state of the field remains inconclusive. It remains unclear whether dermoscopic features of patch test reactions are reaction-specific or primarily reflect reaction intensity, background inflammation, or both. This question may be particularly relevant in AD, where constitutive barrier impairment and persistent inflammation may influence the morphological expression of hapten-induced skin responses. In addition, the available literature remains limited by heterogeneity in study design, assessed dermoscopic parameters, and patient selection, which reduces comparability across studies and contributes to divergent interpretations of similar dermoscopic findings [10,11,12,13].

Another unresolved issue concerns the anatomical compartmentalization of patch test inflammation. The follicular unit represents a distinct immunological niche with antigen-presenting capacity and has been proposed as a potential route of allergen penetration, particularly in barrier-compromised skin [14,15]. Yet it remains unclear whether perifollicular accentuation observed in patch test reactions represents a distinctive marker of AD-associated hapten responsiveness or merely reflects nonspecific background atopic inflammation [16,17].

The present study was conducted as a prospective observational investigation in patients undergoing patch testing for suspected ACD. The primary aim was to characterize the dermoscopic and videodermoscopic features of clinically positive patch test reactions and to compare their morphological expression between individuals with concomitant AD and those without AD. In this context, the study was intended not as a differential diagnostic comparison between AD and ACD as distinct disease entities, but as an assessment of the extent to which concomitant AD may modify the phenotype of allergic patch test reactions. To the best of our knowledge, this is the first prospective study to examine this question systematically in both pediatric and adult populations.

## 2. Materials and Methods

### 2.1. Study Design and Participants

This prospective observational study with repeated assessments was conducted between January 2023 and December 2024 at the Department of Dermatology, Venereology and Allergology, University Clinical Center in Gdańsk, Poland. Consecutive patients referred for routine epicutaneous patch testing were screened for eligibility. For the purposes of the present analysis, the final study cohort was defined a priori by the presence of at least one clinically positive allergic patch test reaction, graded as 1+, 2+, or 3+ according to the International Contact Dermatitis Research Group criteria. Accordingly, the analytical framework of the study was based not on the broad patch-tested population as such but on the subset of individuals with confirmed allergic test responses suitable for dermoscopic evaluation.

The study protocol was approved by the Independent Bioethics Committee for Scientific Research at the Medical University of Gdańsk (approval no. NKKBBN/699/2020; amendment no. NKKBBN/699-218/2021). Written informed consent was obtained from all adult participants. For pediatric participants, written informed consent was obtained from a legal guardian before the initiation of study procedures. The study was conducted in accordance with the Declaration of Helsinki.

Eligible participants were required to be at least 3 years of age, to have a clinical indication for patch testing, to complete all scheduled readings on Days 2, 3, and 7, and to present with at least one clinically positive patch test reaction available for dermoscopic assessment. Because eligibility for the final analytical cohort required completion of all scheduled readings, no missing outcome data were identified within the analyzed cohort. Exclusion criteria comprised generalized dermatitis precluding patch placement, systemic immunosuppressive treatment within four weeks prior to testing, and inability to attend follow-up readings.

Participants were subsequently stratified according to the presence or absence of concomitant AD. The diagnosis of AD was established according to the Hanifin and Rajka criteria and verified at study entry by review of the clinical history and medical records [18]. Participants assigned to the comparison group had no personal history of AD.

The final analytical cohort included 160 participants, comprising 80 patients with AD and 80 patients without AD. Disease severity in the AD group was assessed using the SCORing Atopic Dermatitis (SCORAD) index [19,20] and categorized as mild, moderate, or severe according to conventional thresholds.

All inferential analyses were prespecified at the participant level. Patch test sites and repeated reading time points were used to derive participant-level feature status according to a predefined aggregation rule. This approach was selected a priori to address the clinically relevant question of whether a given dermoscopic feature occurred in an individual undergoing patch testing while avoiding within-participant dependence arising from multiple test sites and repeated assessments.

### 2.2. Patch Testing Procedure

Epicutaneous patch testing was performed using the Polish Baseline Series (Chemotechnique Diagnostics, Vellinge, Sweden). Allergens were applied to the upper back using Finn Chambers^®^ mounted on Scanpor^®^ tape (SmartPractice, Phoenix, AZ, USA), in accordance with routine clinical procedures and the manufacturers’ instructions.

The patches were removed after 48 h (Day 2). Clinical readings were performed at Day 2 (48 h) and Day 3 (72 h), and a late reading was obtained on Day 7 after patch application. Evaluations were performed in accordance with the recommendations of the International Contact Dermatitis Research Group (ICDRG) for diagnostic patch testing [3]. Clinical responses were graded as negative, doubtful (?+), weak positive (1+), strong positive (2+), or extreme positive (3+).

Dermoscopic and videodermoscopic documentation was performed for all patch test sites at each scheduled reading time point (Day 2, Day 3, and Day 7), irrespective of the initial clinical grade. This imaging strategy was prespecified in the study protocol and applied uniformly to all participants. For the primary comparative analysis of dermoscopic morphology, the analytical dataset was restricted a priori to clinically positive patch test reactions (1+, 2+, or 3+) in order to characterize the features of confirmed allergic responses. Clinically doubtful reactions (ICDRG category ?+) were additionally included in the reclassification analysis before and after dermoscopic evaluation. Clinically negative patch test reactions were assessed during routine evaluation but were not included in the formal feature-based comparative analysis.

### 2.3. Dermoscopic and Videodermoscopic Assessment

Dermoscopy of patch test sites was performed at each scheduled reading using a handheld DermLite DL5 dermatoscope (3Gen Inc., San Juan Capistrano, CA, USA) at 15× magnification in polarized and non-polarized modes. High-resolution videodermoscopy was performed using the FotoFinder Vexia system with FotoFinder software (FotoFinder Systems GmbH, Bad Birnbach, Germany), with magnification of up to 140×.

Imaging was performed under standardized conditions. The standard working distance of the device was maintained for all images, and contact pressure was kept minimal and as constant as possible to minimize blanching artifacts. A transparent immersion gel was applied uniformly to all documented sites before contact imaging, and the same gel was used throughout the study. For each documented site and each time point, images were obtained in both polarized and non-polarized modes during handheld dermoscopy and with standardized settings during videodermoscopy.

Dermoscopic evaluation covered the entire reaction area corresponding to the Finn Chamber test field within chamber borders.

The morphological assessment was prespecified and included the following features: homogeneous erythema, perifollicular erythema (perifollicular accentuation), yellowish areas potentially compatible with spongiotic change, vascular pattern, papules/vesicles, scaling, white areas, pigment residuals, and crust formation. Pigment residuals were defined as dermoscopically visible residual exogenous pigment persisting at the patch test site after allergen exposure, most likely reflecting retained exogenous dye-related material rather than non-specific endogenous pigmentation. All features were recorded as binary variables (present/absent) for each documented site at each time point using operational definitions provided in Table S1.

All images were evaluated independently by two assessors, one of whom was a physician board-certified in dermatology and allergology. Image review was performed in a blinded manner. Assessors were blinded to participant group assignment (AD vs. non-AD), clinical patch test grading, SCORAD score, and all other clinical data at the time of dermoscopic assessment. Pre-consensus inter-rater agreement was assessed at the documented site/image level for selected key dermoscopic features using percent agreement and Cohen’s kappa coefficient (listed in Supplementary Table S2). Selected features were those with predefined operational definitions and sufficient prevalence to allow reliable estimation of agreement. Any discrepancies between the two initial assessments were resolved by joint review and consensus, and the consensus-based classification was used for the final analysis.

### 2.4. Outcomes

The primary outcome was the between-group difference (AD vs. non-AD) in the frequency of prespecified dermoscopic and videodermoscopic features among clinically positive patch test reactions, analyzed at the participant level within each age stratum (adults and children). A participant was classified as feature-positive if a given dermoscopic feature was identified in at least one clinically positive patch test site at any scheduled reading (Day 2, Day 3, or Day 7). This participant-level aggregation rule was prespecified and selected to address within-participant non-independence arising from multiple patch test sites and repeated assessments over time. It was intended to capture the participant-level occurrence of a dermoscopic feature rather than its burden across reactions. Accordingly, this binary approach does not retain information on feature frequency, intensity, temporal persistence, or distribution across sites, and participants with a greater number of clinically positive patch test reactions may have had a higher probability of being classified as feature-positive. The findings should therefore be interpreted as participant-level phenotypic differences rather than quantitative differences in dermoscopic feature burden.

Secondary outcomes included associations between selected dermoscopic features and AD severity, as well as the change in the proportion of clinically doubtful patch test reactions (ICDRG category ?+) before and after dermoscopic evaluation. This analysis was intended to assess the potential contribution of dermoscopy to the reclassification of initially uncertain reactions and was not designed to determine the independent diagnostic sensitivity or specificity of dermoscopy across the full spectrum of patch test responses.

### 2.5. Statistical Analysis

All primary analyses were conducted at the participant level within each age stratum (adults and children). For each dermoscopic feature, participants were classified as feature-positive if the feature was observed in at least one clinically positive patch test site at any scheduled reading (Day 2, Day 3, or Day 7). Participant-level analysis was selected a priori because the principal clinical question concerned whether a given dermoscopic feature occurred in a patient undergoing patch testing while also avoiding the statistical dependence introduced by multiple test sites and repeated readings within the same individual. Clinically doubtful and clinically negative patch test reactions were not included in the primary feature-based comparative analysis, which was restricted a priori to clinically positive allergic reactions (1+, 2+, or 3+). Clinically doubtful reactions were, however, included in the reclassification analysis before and after dermoscopic evaluation.

Categorical variables were summarized as counts and percentages. Between-group comparisons (AD vs. non-AD) for each dermoscopic feature within each age stratum were performed using two-sided Fisher’s exact test. Effect sizes were reported as risk ratios (RRs), odds ratios (ORs), and risk differences (RDs) expressed in percentage points (pp), each with 95% confidence intervals (95% CIs). Confidence intervals were calculated using established methods for 2 × 2 tables: RR and OR confidence intervals were computed on the logarithmic scale, whereas RD confidence intervals were estimated using the Newcombe method based on Wilson score intervals.

To account for multiple testing across the prespecified age-stratified primary comparisons reported in Table 1, *p*-values were adjusted using the Benjamini–Hochberg false discovery rate (FDR) procedure; q-values are reported alongside unadjusted *p*-values.

Because dermoscopic and videodermoscopic documentation was performed at all scheduled reading time points for all patch test sites, the comparative analysis reported in this study was based on an a priori defined analytical dataset restricted to clinically positive allergic reactions graded as 1+, 2+, or 3+. Statistical analyses were performed using R software (version 4.3.2; R Foundation for Statistical Computing, Vienna, Austria). 

### 2.6. Availability of Materials, Data, and Protocols

The predefined dermoscopic feature definitions, the study protocol, and the analysis scripts are available. De-identified dermoscopic and videodermoscopic images supporting the findings of this study are available from the corresponding author upon reasonable request, subject to the conditions of ethical approval and applicable data protection regulations. Pre-consensus inter-rater agreement metrics for selected key dermoscopic features are provided in Supplementary Table S1.

### 2.7. Generative AI Statement

No generative artificial intelligence tools were used to generate study data or images, to perform statistical analyses, or to interpret the results.

## 3. Results

### 3.1. Study Population

The final analytical cohort consisted of 160 participants with clinically positive patch test reactions eligible for dermoscopic evaluation, including 80 patients with concomitant AD and 80 patients without AD. These participants were identified from among consecutive individuals undergoing routine epicutaneous patch testing in clinical practice. The AD cohort comprised 37 adults and 43 children, whereas the non-AD cohort included 39 adults and 41 children.

All outcomes reported in this section were analyzed at the participant level within each age stratum (adults/children). A dermoscopic feature was classified as present for a given participant if it was identified in at least one documented clinically positive patch test site at any scheduled reading (Day 2, Day 3, or Day 7).

### 3.2. Dermoscopic Features of Positive Patch Test Reactions in Ad and Non-Ad Participants

Age-stratified analyses demonstrated that several dermoscopic features were more prevalent in patients with AD than in non-AD controls (Table 1). Fisher’s exact test *p*-values are shown together with Benjamini–Hochberg false discovery rate (FDR)-adjusted q-values.

Perifollicular erythema was markedly enriched in children with AD, occurring in 18/43 (41.9%) participants compared with 3/41 (7.3%) non-AD controls. This corresponded to RR 5.72 (95% CI 1.82–17.98), OR 9.12 (95% CI 2.43–34.22), and RD 34.6 percentage points (95% CI 19.0–50.3), with Fisher’s *p* = 3.03 × 10^−4^ and q = 8.08 × 10^−4^. In adults, perifollicular erythema was also more frequent in the AD cohort than in the non-AD cohort, being observed in 13/37 (35.1%) and 6/39 (15.4%) participants, respectively; however, this difference did not reach conventional statistical significance (RR 2.28, 95% CI 0.97–5.38; OR 2.98, 95% CI 0.99–8.96; RD 19.8 percentage points, 95% CI 0.6–38.9; *p* = 0.0643; q = 0.0857).

Yellowish areas, potentially compatible with spongiotic change [21], were consistently more frequent in the AD cohort across both age strata. Among adults, they were present in 16/37 (43.2%) patients with AD and 8/39 (20.5%) non-AD controls, corresponding to RR 2.11 (95% CI 1.03–4.33), OR 2.95 (95% CI 1.07–8.13), and RD 22.7 percentage points (95% CI 2.3–43.1; *p* = 0.0481; q = 0.0770). Among children, yellowish areas were identified in 19/43 (44.2%) patients with AD and 5/41 (12.2%) non-AD controls, corresponding to RR 3.62 (95% CI 1.49–8.80), OR 5.70 (95% CI 1.87–17.34), and RD 32.0 percentage points (95% CI 14.1–49.9; *p* = 1.53 × 10^−3^; q = 3.06 × 10^−3^).

Pigment residuals showed the strongest association with AD across both age strata. In adults, pigment residuals were identified in 26/37 (70.3%) patients with AD compared with 8/39 (20.5%) non-AD controls, corresponding to RR 3.43 (95% CI 1.78–6.58), OR 9.16 (95% CI 3.21–26.16), and RD 49.8 percentage points (95% CI 30.3–69.2; *p* = 2.26 × 10^−5^; q = 1.26 × 10^−4^). In children, the corresponding frequencies were 29/43 (67.4%) and 9/41 (22.0%), yielding RR 3.07 (95% CI 1.66–5.67), OR 7.37 (95% CI 2.77–19.56), and RD 45.5 percentage points (95% CI 26.6–64.4; *p* = 3.15 × 10^−5^; q = 1.26 × 10^−4^).

In contrast, homogeneous erythema was highly prevalent in both cohorts and showed limited discriminatory value. In adults, it was observed in 33/37 (89.2%) patients with AD and 33/39 (84.6%) non-AD controls (*p* = 0.737; q = 0.737). In children, the corresponding frequencies were 39/43 (90.7%) and 35/41 (85.4%), respectively (*p* = 0.515; q = 0.589). Crust formation was not observed in any participant.

**Figure 1:**
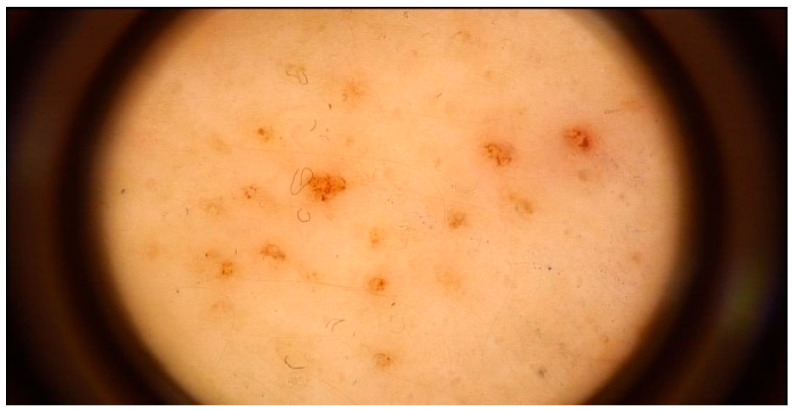
Perifollicular accentuation in a positive patch test reaction in a patient with atopic dermatitis, showing marked perifollicular erythema and scaling. Image acquired by videodermoscopy using the FotoFinder system.

**Figure 2:**
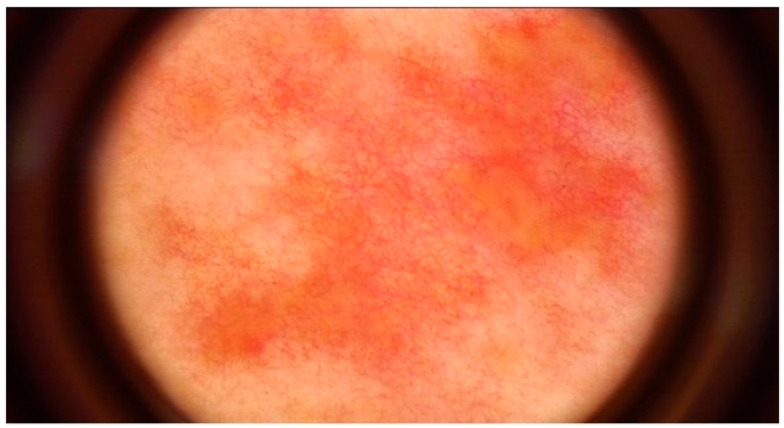
Yellowish structureless areas in a positive patch test reaction in a patient with atopic dermatitis. Image acquired by videodermoscopy using the FotoFinder system (FotoFinder Systems GmbH, Bad Birnbach, Germany).

**Figure 3:**
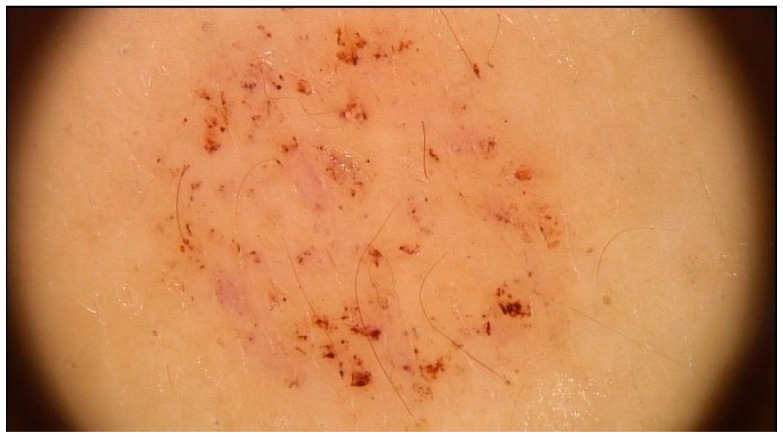
Pigment residuals appearing as focal brownish deposits within an inflamed positive patch test reaction in a patient with atopic dermatitis. Image acquired by videodermoscopy using the FotoFinder system.

**Figure 4:**
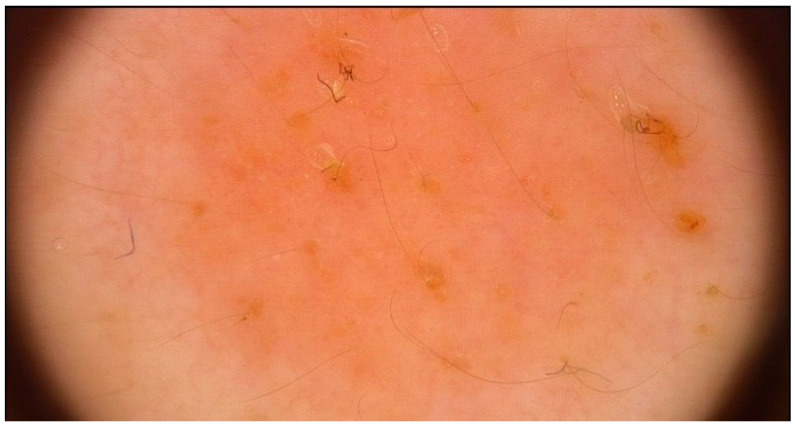
Diffuse homogeneous erythema in a positive patch test reaction, observed in patients with and without atopic dermatitis and showing limited discriminatory value. Image acquired by videodermoscopy using the FotoFinder system.

Representative dermoscopic findings are illustrated in Figure 1, Figure 2, Figure 3 and Figure 4. Perifollicular accentuation, yellowish structureless areas, and pigment residuals illustrate the principal dermoscopic features enriched in AD-associated reactions (Figure 1, Figure 2 and Figure 3), whereas diffuse homogeneous erythema was observed in both cohorts and therefore demonstrated limited discriminatory utility (Figure 4).

Collectively, these findings suggest that clinically positive patch test reactions in patients with AD are characterized primarily by increased frequencies of pigment residuals and yellowish areas across both age strata, with perifollicular erythema emerging as an additional distinguishing feature, particularly in children.

### 3.3. Association Between Dermoscopic Findings and Ad Severity

Within the AD cohort, yellowish areas were associated with greater disease severity as assessed by SCORAD. Among adults, yellowish areas were observed in 11/13 patients with moderate AD compared with 5/24 patients with mild AD (*p* < 0.001). Among children, this feature was present in all patients with moderate AD (14/14) but only in 5/29 patients with mild AD (*p* < 0.00001). Overall, these results indicate enrichment of dermoscopic patterns potentially compatible with greater epidermal involvement among participants with more severe AD.

### 3.4. Pre-Consensus Inter-Rater Agreement

Pre-consensus inter-rater agreement for selected key dermoscopic features was moderate to almost perfect across study strata. Percent agreement ranged from 83.8% to 97.4%, while Cohen’s kappa values ranged from 0.55 to 0.89. Agreement varied across features and study strata, with the highest values observed for homogeneous erythema and pigment residuals. Detailed agreement metrics for each feature and study stratum are provided in Supplementary Table S1.

### 3.5. Reclassification of Clinically Doubtful Reactions After Dermoscopic Evaluation

Macroscopic assessment prior to dermoscopy yielded a higher proportion, a substantially higher proportion, of clinically doubtful patch test reactions (ICDRG category ?+) in patients with AD than in non-AD controls (Table 2). Following dermoscopic evaluation, the proportion of clinically doubtful reactions decreased across all strata, with the largest absolute reductions observed in the AD cohort.

As shown in Table 2, dermoscopy was associated with a reduction in the proportion of clinically doubtful reactions compared with macroscopic assessment. These findings should not be interpreted as evidence of improved diagnostic accuracy, but rather as reflecting a change in morphological interpretation following dermoscopic assessment. In the non-AD cohort, doubtful results decreased from 33.3% (13/39) to 15.4% (6/39) in adults and from 26.8% (11/41) to 7.3% (3/41) in children. In the AD cohort, the reductions were substantially larger, from 73.0% (27/37) to 13.5% (5/37) in adults and from 58.1% (25/43) to 4.7% (2/43) in children. As shown in Table 2, reactions initially classified as clinically positive on routine macroscopic assessment were more frequently downgraded or otherwise reclassified after dermoscopic evaluation in the AD cohort than in the non-AD cohort. Among adults, such reclassification occurred in 25/37 (67.6%) patients with AD compared with 7/39 (17.9%) non-AD controls, corresponding to RR 3.76 (95% CI 1.86–7.64) and OR 9.52 (95% CI 3.27–27.74; Fisher’s *p* = 1.94 × 10^−5^). Among children, the corresponding frequencies were 33/43 (76.7%) and 10/41 (24.4%), respectively, yielding RR 3.15 (95% CI 1.79–5.53) and OR 10.23 (95% CI 3.75–27.93; *p* = 2.79 × 10^−6^). In the total sample, reclassification after dermoscopic evaluation occurred in 58/80 (72.5%) patients with AD and 17/80 (21.2%) non-AD controls, corresponding to RR 3.41 (95% CI 2.19–5.31) and OR 9.77 (95% CI 4.72–20.20; *p* = 9.03 × 10^−11^).

## 4. Discussion

The present study demonstrates that clinically positive patch test reactions arising in patients with atopic dermatitis exhibit a consistent dermoscopic pattern, characterized predominantly by perifollicular accentuation, yellowish structureless areas, and persistent pigment residuals, whereas homogeneous erythema alone offers limited discriminatory value. Importantly, these findings should be interpreted within the specific framework of confirmed allergic patch test responses rather than as a direct comparison between AD and allergic contact dermatitis as disease entities. Instead, our results suggest that the atopic skin background acts as a biologically relevant modifier of allergen-induced reaction morphology, thereby influencing how positive patch test responses are visually expressed and, potentially, clinically interpreted [10,11,22].

This interpretation is biologically plausible. AD is characterized by epidermal barrier dysfunction, altered keratinocyte differentiation, and predominantly type 2-skewed inflammation [23]. Allergic contact dermatitis, in contrast, represents an antigen-specific delayed-type hypersensitivity reaction elicited after sensitization [4,24,25]. Patch test reactions arising in atopic skin therefore develop within tissue that is already structurally compromised and immunologically activated. Their visible morphology is consequently likely to reflect not only hapten-induced immune activation, but also the pre-existing atopic cutaneous milieu.

An important implication of our findings is that patch test reactions in AD should not be interpreted simply as stronger versions of those observed in non-AD skin. Rather, AD appears to modify the micro-morphological organization of the inflammatory response itself. This is particularly evident in the behavior of homogeneous erythema. Although erythema remains the dominant visible endpoint in conventional patch test scoring, it is also a common baseline feature of atopic skin, related to persistent inflammation and altered vascular reactivity [3,4,21]. Its high prevalence in both cohorts and poor discriminatory performance in the present study therefore expose a central limitation of erythema-based interpretation in AD. Dermoscopy addresses this limitation by shifting evaluation from non-specific redness toward more discrete and reproducible structural features [10,11,17].

Perifollicular accentuation emerged as a particularly informative feature, especially in the pediatric AD subgroup. This finding is consistent with the biological role of the follicular unit as an active epidermal compartment and an important route for the penetration of topically applied substances [14,15,26,27]. In barrier-deficient skin, follicular pathways may facilitate local allergen entry and amplify compartmentalized inflammation, making perifollicular erythema a plausible dermoscopic correlate of spatially organized immune activation [28]. From a clinical perspective, this feature may be underestimated on routine inspection, particularly in weak or borderline reactions, yet becomes readily appreciable under dermoscopy. Perifollicular accentuation may therefore provide a useful indicator of how inflammatory responses are anatomically organized in atopic skin.

Yellowish structureless areas were significantly more frequent in the AD cohort and were associated with greater disease severity as assessed by SCORAD. This observation suggests that dermoscopic morphology may capture clinically meaningful differences in epidermal inflammatory change linked to more active atopic disease. In barrier-impaired skin exposed to epicutaneous challenge, such reactions may be more likely to assume a spongiotic pattern; however, in the absence of histopathological confirmation, the interpretation of yellowish areas as reflecting spongiotic change should be regarded as biologically plausible but inferential. Accordingly, these findings are better understood as indicating a possible relationship between yellowish dermoscopic morphology and greater epidermal involvement in active AD, rather than as direct proof of a specific underlying histopathological substrate [4,5,29,30,31].

Pigment residuals showed the strongest association with AD in both adults and children. In the present study, this feature denotes dermoscopically visible residual exogenous pigment persisting at the patch test site after allergen exposure, rather than non-specific endogenous post-inflammatory pigmentation. The observed distribution is consistent with this interpretation. Among the 72 reactions in which pigment residuals were identified, most were associated with dye-related allergens, particularly para-phenylenediamine (PPD) (47/72, 65.3%) and textile dye mix (23/72, 31.9%), whereas chromium contributed only marginally (2/72, 2.8%). Accordingly, one possible interpretation is that pigment residuals reflect pigment retention in chronically inflamed atopic skin [4,5,6,21,32]. Nevertheless, because no histopathological examination or analytical chemical characterization was performed, the exact molecular composition of the retained pigment, including its specific derivatives, cannot be established with certainty.

Beyond phenotypic characterization, our findings suggest that dermoscopy may provide additional morphological information relevant to the interpretation of uncertain patch test reactions. In the present study, dermoscopic evaluation was associated with a reduction in the proportion of clinically doubtful reactions, particularly in patients with AD, in whom reactive skin and subtle inflammatory changes may complicate routine visual assessment [8,9,10,17,33]. These observations should, however, be interpreted in light of the study design. Although clinically doubtful reactions were included in the reclassification analysis and clinically negative reactions were systematically examined, the primary feature-based comparative analysis focused on clinically positive reactions, and the study was not designed as a formal diagnostic accuracy analysis. Accordingly, the present findings support the adjunctive morphological value of dermoscopy and its association with reclassification of clinically doubtful reactions, rather than establishing its independent diagnostic performance across the full spectrum of patch test responses.

From a clinical standpoint, dermoscopy should be regarded as an adjunct rather than a replacement for conventional patch test reading. Its greatest utility is likely to lie in weak or borderline reactions, in patients with pronounced background erythema, and in situations where pigmentary change or structural heterogeneity complicates later readings [10,11,17]. Notably, the clinical interpretability of these dermoscopic findings was strengthened by their reproducibility across independent assessors: pre-consensus inter-rater agreement for key features ranged from 83.8% to 97.4%, with Cohen’s κ values of 0.55–0.89, consistent with moderate to almost perfect agreement (Supplementary Table S1).

Several limitations should be acknowledged. First, this was a single-center study, which may limit generalizability. Second, although image assessment was standardized and based on predefined criteria, dermoscopic interpretation remains at least partly observer-dependent despite the moderate to almost perfect pre-consensus agreement observed for selected key features. Third, the primary feature-based comparative analysis was restricted to clinically positive patch test reactions and conducted at the participant level, which strengthened interpretive consistency but may have reduced sensitivity to site-specific heterogeneity. A further limitation relates to the participant-level aggregation strategy, whereby a dermoscopic feature was considered present if it was identified at least once in any clinically positive patch test site at any scheduled reading. This approach was selected to avoid within-participant dependence and to address the clinically relevant question of whether a given feature occurred at all in a patient. However, it does not retain information on the number of affected sites, feature persistence across time points, or feature intensity. Therefore, the observed between-group differences should be interpreted as participant-level phenotypic contrasts rather than quantitative differences in dermoscopic burden. Moreover, if one group had a greater number of clinically positive patch test sites overall, the probability of fulfilling the “present at least once” criterion may also have been higher. Therefore, the potential influence of differences in the number of clinically positive patch test reactions per participant cannot be fully excluded. Finally, the study did not include histopathological validation. Therefore, direct mapping between dermoscopic morphology and specific underlying biological processes cannot be established on the basis of the present data. Any proposed links between features such as yellowish areas or pigment residuals and processes such as spongiotic change or chronic inflammatory remodeling should be regarded as biologically plausible but inferential and hypothesis-generating rather than confirmatory.

Taken together, the present findings suggest that hapten-induced reactions in AD develop on a cutaneous background that differs structurally and functionally from that of non-AD skin, and that these differences may be reflected in dermoscopic morphology. At the same time, proposed links between dermoscopic patterns and underlying biological processes should be interpreted as biologically informed hypotheses rather than direct evidence of specific histopathological mechanisms.

## 5. Conclusions

Positive patch test reactions in patients with atopic dermatitis may display a consistent dermoscopic pattern with potential clinical relevance, characterized by perifollicular accentuation, yellowish areas potentially compatible with spongiotic change, and pigment residuals. Dermoscopy may provide adjunctive morphological support for patch test interpretation, particularly in clinically doubtful reactions, but its independent diagnostic performance was not assessed in the present study.

## Figures and Tables

**Table 1:** Dermoscopic features of clinically positive patch test reactions in participants with and without concomitant atopic dermatitis (AD), stratified by age group (participant-level analysis).

Feature	Age Group	AD n/N (%)	Non-AD, n/N (%)	RR (95% CI)	OR (95% CI)	RD, pp (95% CI)	Fisher’s *p*	q (FDR)
Perifollicular erythema	Adults	13/37 (35.1%)	6/39 (15.4%)	2.28 (0.97–5.38)	2.98 (0.99–8.96)	19.8 (0.6–38.9)	0.0643	0.0857
Perifollicular erythema	Children	18/43 (41.9%)	3/41 (7.3%)	5.72 (1.82–17.98)	9.12 (2.43–34.22)	34.6 (19.0–50.3)	3.03 × 10^−4^	8.08 × 10^−4^
Yellowish areas	Adults	16/37 (43.2%)	8/39 (20.5%)	2.11 (1.03–4.33)	2.95 (1.07–8.13)	22.7 (2.3–43.1)	0.0481	0.0770
Yellowish areas	Children	19/43 (44.2%)	5/41 (12.2%)	3.62 (1.49–8.80)	5.70 (1.87–17.34)	32.0 (14.1–49.9)	1.53 × 10^−3^	3.06 × 10^−3^
Pigment residuals	Adults	26/37 (70.3%)	8/39 (20.5%)	3.43 (1.78–6.58)	9.16 (3.21–26.16)	49.8 (30.3–69.2)	2.26 × 10^−5^	1.26 × 10^−4^
Pigment residuals	Children	29/43 (67.4%)	9/41 (22.0%)	3.07 (1.66–5.67)	7.37 (2.77–19.56)	45.5 (26.6–64.4)	3.15 × 10^−5^	1.26 × 10^−4^
Homogeneous erythema	Adults	33/37 (89.2%)	33/39 (84.6%)	1.05 (0.89–1.26)	1.50 (0.39–5.81)	4.6 (−10.5–19.7)	0.737	0.737
Homogeneous erythema	Children	39/43 (90.7%)	35/41 (85.4%)	1.06 (0.91–1.25)	1.67 (0.44–6.41)	5.3 (−8.5–19.2)	0.515	0.589

Abbreviations: AD, atopic dermatitis; non-AD, participants without atopic dermatitis; CI, confidence interval; FDR, false discovery rate; OR, odds ratio; pp, percentage points; RD, risk difference; RR, risk ratio.

**Table 2:** Effect of dermoscopic evaluation on reclassification of patch test reactions in participants with and without atopic dermatitis (AD), stratified by age group (participant-level analysis).

Panel A. Reduction of Clinically Doubtful (?+) Reactions Before vs. After Dermoscopy					
Cohort	Age group	Clinically doubtful before n/N (%)	Clinically doubtful after n/N (%)		Absolute reduction, pp
Non-AD	Adults	13/39 (33.3)	6/39 (15.4)		17.9
Non-AD	Children	11/41 (26.8)	3/41 (7.3)		19.5
AD	Adults	27/37 (73.0)	5/37 (13.5)		59.5
AD	Children	25/43 (58.1)	2/43 (4.7)		53.4
Panel B. Initially clinically positive patch test reactions reclassified after dermoscopic evaluation					
**Age group**	**AD n/N (%)**	**non-AD n/N (%)**	**RR (95% CI)**	**OR (95% CI)**	**Fisher’s *p***
Adults	25/37 (67.6)	7/39 (17.9)	3.76 (1.86–7.64)	9.52 (3.27–27.74)	1.94 × 10^−5^
Children	33/43 (76.7)	10/41 (24.4)	3.15 (1.79–5.53)	10.23 (3.75–27.93)	2.79 × 10^−6^
Total	58/80 (72.5)	17/80 (21.2)	3.41 (2.19–5.31)	9.77 (4.72–20.20)	9.03 × 10^−11^

Abbreviations: AD, atopic dermatitis; non-AD, participants without atopic dermatitis; CI, confidence interval; OR, odds ratio; pp, percentage points; RR, risk ratio.

## Data Availability

The data presented in this study are not publicly available due to privacy and ethical restrictions but are available from the corresponding author upon reasonable request and subject to approval by the relevant ethics committee.

## Supplementary Materials

The following supporting information can be downloaded at: https://www.mdpi.com/article/10.3390/life16040663/s1, Table S1. Pre-consensus inter-rater agreement for selected dermoscopic features.

## Abbreviations

The following abbreviations are used in this manuscript:

ACD	Allergic contact dermatitis
AD	Atopic dermatitis
CI	Confidence interval
ICDRG	International Contact Dermatitis Research Group
OR	Odds ratio
pp	Percentage points
RD	Risk difference
RR	Risk ratio
SCORAD	SCORing Atopic Dermatitis
FDR	False discovery rate

## References

[1] Nosbaum A., Vocanson M., Rozieres A., Hennino A., Nicolas J.F. (2009). Allergic and irritant contact dermatitis. Eur. J. Dermatol..

[2] Mahler V., Nast A., Bauer A., Becker D., Brasch J., Breuer K., Dickel H., Drexler H., Elsner P., Geier J. (2019). S3 Guidelines: Epicutaneous patch testing with contact allergens and drugs—Short version, Part 2. J. Dtsch. Dermatol. Ges..

[3] Johansen J.D., Aalto-Korte K., Agner T., Andersen K.E., Bircher A., Bruze M., Cannavó A., Giménez-Arnau A., Gonçalo M., Goossens A. (2015). European Society of Contact Dermatitis guideline for diagnostic patch testing—Recommendations on best practice. Contact Dermat..

[4] Brunner P.M., Guttman-Yassky E., Leung D.Y. (2017). The immunology of atopic dermatitis and its reversibility with broad-spectrum and targeted therapies. J. Allergy Clin. Immunol..

[5] Beck L.A., Cork M.J., Amagai M., De Benedetto A., Kabashima K., Hamilton J.D., Rossi A.B. (2022). Type 2 inflammation contributes to skin barrier dysfunction in atopic dermatitis. JID Innov..

[6] Kim B.E., Leung D.Y.M. (2018). Significance of skin barrier dysfunction in atopic dermatitis. Allergy Asthma Immunol. Res..

[7] Scharschmidt T.C., Man M.Q., Hatano Y., Crumrine D., Gunathilake R., Sundberg J.P., Silva K.A., Mauro T.M., Hupe M., Cho S. (2009). Filaggrin deficiency confers a paracellular barrier abnormality that reduces inflammatory thresholds to irritants and haptens. J. Allergy Clin. Immunol..

[8] Fonacier L. (2015). A practical guide to patch testing. J. Allergy Clin. Immunol. Pract..

[9] Le Coz C.J., Sasseville D. (2009). Interprétation et pertinence des patch-tests: Faux-positifs et faux-négatifs, allergies composées, allergies croisées. Ann. Dermatol. Venereol..

[10] Oppermann K., Cattani C.A.S., Bonamigo R.R. (2021). Usefulness of dermoscopy in the evaluation of patch test reactions. An. Bras. Dermatol..

[11] Corazza M., Toni G., Scuderi V., Forconi R., Borghi A. (2019). Patch test reactions through the lens of dermoscopy: Further insights, particularly on weak allergic reactions. Contact Dermat..

[12] Errichetti E., Stinco G. (2016). Dermoscopy in general dermatology: A practical overview. Dermatol. Ther..

[13] Lazzarini R., Duarte I., Ferreira A.L. (2013). Patch tests. An. Bras. Dermatol..

[14] Tordesillas L., Lozano-Ojalvo D., Dunkin D., Mondoulet L., Agudo J., Merad M., Sampson H.A., Berin M.C. (2018). PDL2+ CD11b+ dermal dendritic cells capture topical antigen through hair follicles to prime LAP+ Tregs. Nat. Commun..

[15] Moresi J.M., Horn T.D. (1997). Distribution of Langerhans cells in human hair follicle. J. Cutan. Pathol..

[16] Nedorost S.T., Babineau D. (2010). Patch testing in atopic dermatitis. Dermatitis.

[17] Gil-Pallares P., Navarro-Bielsa A., González-Ruiz A.A., Silvestre J.F. (2023). Dermoscopy in patch testing: How can it help?. Actas Dermosifiliogr..

[18] Hanifin J.M., Rajka G. (1980). Diagnostic Features of Atopic Dermatitis. Acta Derm. Venereol..

[19] Stadler J.F. (1993). Severity scoring of atopic dermatitis: The SCORAD index. Consensus Report of the European Task Force on Atopic Dermatitis. Dermatology.

[20] Kunz B., Oranje A.P., Labrèze L., Stalder J.F., Ring J., Taïeb A. (1997). Clinical validation and guidelines for the SCORAD index: Consensus report of the European Task Force on Atopic Dermatitis. Dermatology.

[21] Bhatt M.M., Jamale V., Hussain A.A., Ankad B.S., Nikam B.P., Kale M., Shelke S.S. (2023). An observational study of dermoscopic and histopathological correlation in spongiotic disorders—A hospital-based cross-sectional study. Indian J. Dermatol..

[22] Lallas A., Zalaudek I., Argenziano G., Longo C., Moscarella E., Di Lernia V., Al Jalbout S., Apalla Z. (2013). Dermoscopy in general dermatology. Dermatol. Clin..

[23] Beltrani V.S. (1999). The clinical spectrum of atopic dermatitis. J. Allergy Clin. Immunol..

[24] Goleva E., Berdyshev E., Leung D.Y. (2019). Epithelial barrier repair and prevention of allergy. J. Clin. Investig..

[25] Tramontana M., Hansel K., Bianchi L., Sensini C., Malatesta N., Stingeni L. (2023). Advancing the understanding of allergic contact dermatitis: From pathophysiology to novel therapeutic approaches. Front. Med..

[26] Lademann J., Knorr F., Richter H., Blume-Peytavi U., Vogt A., Antoniou C., Sterry W., Patzelt A. (2008). Hair follicles—An efficient storage and penetration pathway for topically applied substances. Summary of recent results obtained at the Center of Experimental and Applied Cutaneous Physiology, Charité–Universitätsmedizin Berlin, Germany. Ski. Pharmacol. Physiol..

[27] Kiselev A., Park S. (2024). Immune niches for hair follicle development and homeostasis. Front. Physiol..

[28] Rahmani W., Sinha S., Biernaskie J. (2020). Immune modulation of hair follicle regeneration. NPJ Regen. Med..

[29] Tanei R., Hasegawa Y. (2022). Immunological pathomechanisms of spongiotic dermatitis in skin lesions of atopic dermatitis. Int. J. Mol. Sci..

[30] Soter N.A. (1989). Morphology of atopic eczema. Allergy.

[31] Marsella R., Olivry T., Maeda S. (2006). Cellular and cytokine kinetics after epicutaneous allergen challenge (atopy patch testing) with house dust mites in high-IgE beagles. Vet. Dermatol..

[32] Tsakok T., Woolf R., Smith C.H., Weidinger S., Flohr C. (2019). Atopic dermatitis: The skin barrier and beyond. Br. J. Dermatol..

[33] Brasch J., Schnuch A., Uter W. (2003). Patch-test reaction patterns in patients with a predisposition to atopic dermatitis. Contact Dermat..

