# Supplementary material for: Influence of Atopic Dermatitis on the Dermoscopic Phenotype of Positive Patch Test Reactions: A Prospective Comparative Study
Source: Life (Basel). 2026 Apr 14;16(4):663. doi: 10.3390/life16040663 (PMC13117260; doi:10.3390/life16040663)
Supplement: Supplementary file 1 [file life-16-00663-s001.zip › life-4226586-supplementary.pdf]

Supplementary Table S1. Pre-consensus inter-rater agreement for selected dermoscopic features.

Feature	Age Group	Group	Percent Agreement (%)	Cohen's Kappa (κ)
Perifollicular erythema	Children	AD	86.0	0.72
Perifollicular erythema	Children	Non-AD	90.7	0.55
Perifollicular erythema	Adults	AD	91.9	0.83
Perifollicular erythema	Adults	Non-AD	83.8	0.56
Yellowish areas	Children	AD	88.4	0.77
Yellowish areas	Children	Non-AD	87.8	0.59
Yellowish areas	Adults	AD	86.5	0.73
Yellowish areas	Adults	Non-AD	92.3	0.79
Pigment residuals	Children	AD	93.0	0.83
Pigment residuals	Children	Non-AD	92.7	0.81
Pigment residuals	Adults	AD	94.6	0.86
Pigment residuals	Adults	Non-AD	92.3	0.79
Homogeneous erythema	Children	AD	95.3	0.64
Homogeneous erythema	Children	Non-AD	95.1	0.77
Homogeneous erythema	Adults	AD	97.3	0.84
Homogeneous erythema	Adults	Non-AD	97.4	0.89

Abbreviations: AD, atopic dermatitis; non-AD, participants without atopic dermatitis; κ, Cohen s kappa.
